# Healthy longevity in a mobile world: expatriates as quasi-experimental populations for understanding life-course transitions.

**DOI:** 10.7189/jogh.16.03044

**Published:** 2026-11-13

**Authors:** Angella Anjimile Katenga Maliakini, Krit Pongpirul

**Affiliations:** 1 Graduate Program in Clinical Sciences, Chulalongkorn University Faculty of Medicine, Bangkok, Thailand; 2 Clinical Research Center, Bumrungrad International Hospital, Bangkok, Thailand; 3 Center of Excellence in Preventive and Integrative Medicine (CE-PIM), Chulalongkorn University Faculty of Medicine, Bangkok, Thailand; 4 School of Global Health, Chulalongkorn University Faculty of Medicine, Bangkok, Thailand; 5 Department of Preventive and Social Medicine, Chulalongkorn University Faculty of Medicine, Bangkok, Thailand; 6 Department of Infection Biology & Microbiomes, University of Liverpool Faculty of Health and Life Sciences, Liverpool, UK

**Keywords:** healthy longevity, life-course epidemiology, expatriates, migration and health, global mobility, healthspan

## Abstract

Healthy longevity frameworks increasingly emphasise life-course perspectives, prevention, and the maintenance of functional ability across ageing. However, most existing models implicitly assume populations are embedded within stable national contexts and insufficiently account for the growing role of international mobility. Expatriates experience repeated transitions across healthcare systems, sociocultural environments, and physical contexts that may influence healthy longevity trajectories over time. We propose a conceptual framework for understanding healthy longevity in a mobile world, drawing on purposively selected literature from life-course epidemiology, migration and health, healthy longevity, and health systems research to develop a hypothesis-generating conceptual framework. The framework integrates established concepts from life-course epidemiology with contemporary perspectives on migration as a dynamic and multistage process characterised by changing exposures across origin, destination, and return settings. We propose that repeated geographic transitions represent important life-course inflection points that may reshape healthcare system exposures, sociocultural and behavioural contexts, and psychosocial and environmental conditions. These cumulative exposures may influence awareness, knowledge, motivation, preventive engagement, and continuity of care across systems, thereby affecting pathways associated with mental health, cardiometabolic risk, immune regulation, functional ability, and healthy longevity. The framework further identifies potential modifiers, including age, timing of migration, sex and gender, socioeconomic position, insurance coverage, legal status, digital health infrastructure, and social support. Finally, we generated several empirically testable propositions to guide future longitudinal, comparative, and mixed-method research. We propose that expatriates may serve as quasi-experimental populations for understanding how mobility and life-course transitions shape healthy longevity. Integrating mobility into conceptual and empirical models of healthy ageing may improve the contextual relevance, implementation, and global applicability of interventions aimed at extending healthspan in increasingly interconnected societies.

Global life expectancy has increased substantially over recent decades, driven by advances in public health, medical care, and socioeconomic development. However, gains in lifespan have not been accompanied by equivalent improvements in healthspan, resulting in prolonged periods of chronic disease, functional decline, and reduced quality of life [1]. Consequently, contemporary healthy longevity frameworks increasingly emphasise prevention, life-course perspectives, and the optimisation of health trajectories across time [2,3]. Healthy longevity is increasingly viewed not merely as survival, but as the maintenance of functional ability through interactions among intrinsic capacity and the physical and social environments experienced throughout life.

Life-course epidemiology recognises that health trajectories arise from the accumulation of biological, behavioural, socioeconomic, and environmental exposures across different stages of life [2,4,5]. Yet most healthy longevity models implicitly assume populations embedded within relatively stable national contexts. In reality, contemporary mobility has become increasingly common and should be viewed not as a single event, but as a dynamic and multistage process encompassing repeated transitions across origin, destination, and return settings [6,7]. More than 280 million people currently live outside their country of birth, highlighting the scale at which geographic transitions may influence health trajectories [8].

Migration and international mobility create naturally occurring variation in healthcare systems, sociocultural environments, social relationships, and preventive care practices. Previous research has suggested that migration offers quasi-experimental and counterfactual scenarios that may help disentangle the effects of environmental and behavioural exposures across the life course [9]. Cross-border health-seeking behaviours are further shaped by differences in availability, affordability, familiarity, and perceived quality of healthcare services [10], while social integration itself has been shown to exert substantial effects on survival [11]. These observations suggest that mobility may act as an important modifier of healthy longevity trajectories.

Expatriates – individuals residing abroad for work, retirement, or long-term settlement – represent a strategically important population in which these processes are particularly apparent. Their transnational life courses involve repeated exposure to diverse healthcare systems, cultures, and social environments that are not fully captured within conventional single-country frameworks. Rather than viewing mobility as a deviation from otherwise stable life trajectories, integrating mobility and life-course transitions into healthy longevity research may provide a more globally relevant framework for understanding how changing contextual exposures shape healthspan in an increasingly interconnected world.

## EXPATRIATES AS QUASI-EXPERIMENTAL POPULATIONS

Expatriates occupy a unique position at the intersection of multiple healthcare, sociocultural, and environmental systems. Over time, they encounter differing healthcare infrastructures, insurance models, regulatory environments, and societal attitudes toward ageing and prevention. Consistent with life-course epidemiology, these exposures accumulate across time and may be modified by repeated relocations, giving rise to dynamic and context-dependent health trajectories. Migration itself is increasingly recognised as a multistage and cyclical process, rather than a single event, encompassing changing exposures across origin, destination, and return settings [6,7].

Migration research has described the ‘healthy migrant effect’, whereby migrant populations may initially exhibit more favourable health profiles than host populations [12]. However, the magnitude and persistence of this phenomenon are heterogeneous and vary according to migrant category, country of origin, host setting, and socioeconomic context. These observations should, therefore, not be directly extrapolated to expatriates. Rather, they illustrate a broader point: mobility-related health trajectories are dynamic and context-dependent rather than fixed. Such trajectories may evolve through interactions among healthcare access, sociocultural adaptation, environmental exposures, and life-course experiences [9,12].

Although migration and health have been extensively studied, much of the literature has focused on cultural adjustment, occupational stress, mental health, and healthcare access [13,14]. Comparatively less attention has been directed toward healthy longevity and life-course dimensions among expatriate populations. Furthermore, individuals may actively navigate multiple healthcare ecosystems and seek care across borders according to differences in availability, affordability, familiarity, and perceived quality of services [10].

Expatriates are highly heterogeneous with respect to socioeconomic position, insurance coverage, legal residency status, and available resources. Consequently, the effects of mobility on healthy longevity are unlikely to be uniform. Corporate expatriates with employer-sponsored benefits and guaranteed repatriation arrangements may experience greater continuity of care and broader healthcare choices than retirees living on fixed incomes or individuals with more precarious residency circumstances. Socioeconomic privilege may, therefore, mediate how mobility translates into healthcare access, preventive engagement, and health trajectories across the life course.

Previous migration scholarship has suggested that migration provides naturally occurring ‘quasi-experimental and counterfactual scenarios’ that facilitate the study of environmental and behavioural influences on health across the life course [9,12]. In this sense, expatriates may be viewed as quasi-experimental populations whose experiences reveal how mobility, contextual variability, and repeated system transitions interact to shape health perceptions, preventive behaviours, and healthy longevity trajectories in ways that are less apparent in geographically stable populations.

## CONCEPTUAL FRAMEWORK DEVELOPMENT

We developed this viewpoint through a purposive review of literature from life-course epidemiology, migration and health, healthy longevity, and health systems research. Rather than undertaking a systematic or scoping review, we selected literature to illustrate key theoretical concepts and empirical observations relevant to mobility and healthy longevity.

The framework we propose based on this literature integrates established concepts from life-course epidemiology, including latency, cumulative effects, and pathway mechanisms, with contemporary migration-health perspectives that conceptualise migration as a dynamic and multistage process involving repeated transitions across origin, destination, and return settings. As such, it is intended to generate hypotheses for future empirical investigation, rather than to represent a validated causal model.

## HYPOTHESIS-GENERATING LIFE-COURSE FRAMEWORK

Healthy longevity emerges from the accumulation of biological, behavioural, socioeconomic, and environmental exposures across the life course. Consistent with life-course epidemiology, health trajectories reflect latency, cumulative effects, and pathway mechanisms operating across different stages of life [2,5]. We propose that repeated geographic transitions represent important inflection points that may modify these trajectories over time (**Figure 1**). For expatriates, relocation dynamically reshapes healthcare systems, sociocultural and behavioural environments, and psychosocial and environmental contexts across different life stages.

**Figure 1:**
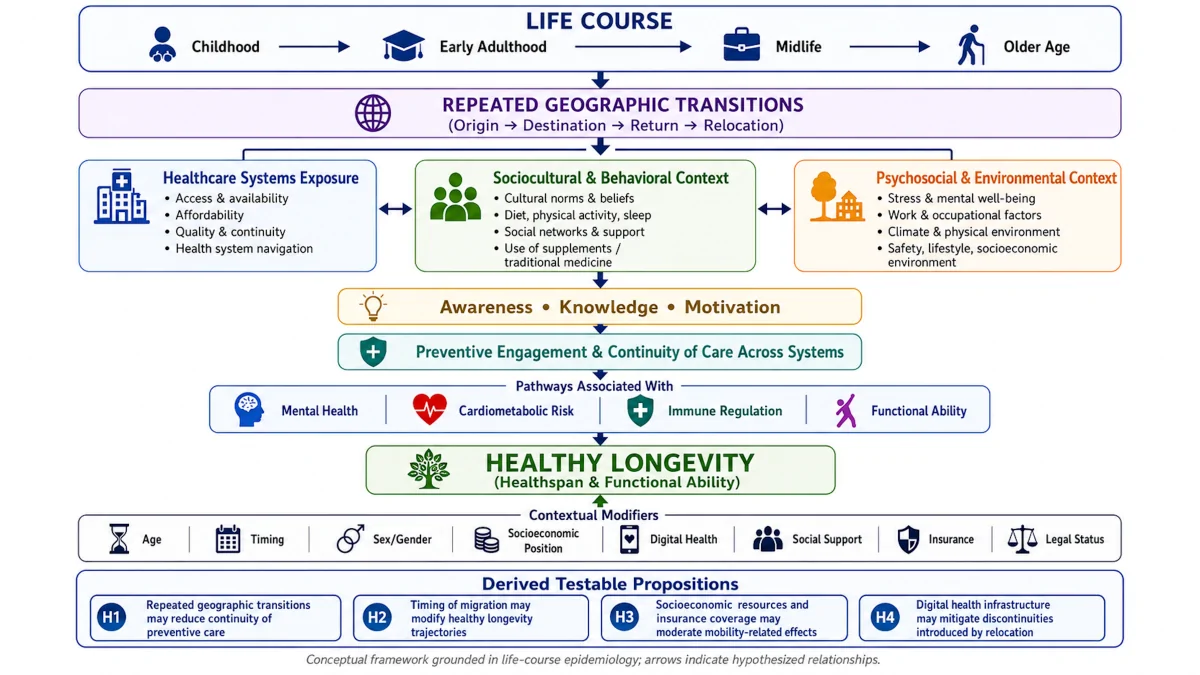
Hypothesis-generating life-course framework of expatriate healthy longevity. Repeated geographic transitions are conceptualised as life-course inflection points that may reshape healthcare system exposures, sociocultural and behavioural contexts, and psychosocial and environmental contexts. These contextual exposures may influence awareness, knowledge, motivation, preventive engagement, and continuity of care across systems, thereby shaping pathways associated with mental health, cardiometabolic risk, immune regulation, functional ability, and healthy longevity. The framework also highlights contextual modifiers, including age, timing of migration, sex and gender, socioeconomic position, digital health, social support, insurance coverage, and legal status. The proposed framework is conceptual, rather than causal, and is intended to generate empirically testable propositions for future longitudinal, comparative, and mixed-method studies.

First, movement across healthcare systems may influence healthcare utilisation, screening uptake, continuity of care, and engagement with preventive interventions. Individuals frequently navigate multiple healthcare ecosystems and may seek care across borders according to differences in availability, affordability, familiarity, and perceived quality of services [10]. Variations in accessibility, financing structures, and emphasis on prevention may alter trust in providers and long-term health-seeking behaviour [15].

Second, expatriates encounter changing sociocultural environments that shape beliefs, norms, and daily health practices. Language, social relationships, and cultural expectations influence diet, physical activity, sleep, healthcare utilisation, and the use of supplements or traditional medicine. Over time, many individuals develop hybrid behavioural patterns that integrate practices from both origin and host cultures [16]. Social integration and support may further influence health trajectories through behavioural, psychological, and biological pathways [11].

Third, geographic transitions are often accompanied by psychosocial and environmental stressors, including acculturation challenges, social isolation, occupational pressures, climate-related exposures, and altered food environments. These factors may influence mental health and cardiometabolic risk and may also affect pathways associated with immune regulation and biological ageing [17]. The magnitude and direction of these effects are likely to vary according to age, sex, gender, socioeconomic position, and timing of migration across the life course [18,19].

Mobility-related health trajectories are also likely to differ according to biological sex and sociocultural gender. Repeated relocations may intersect with reproductive transitions, including pregnancy, childbirth, and menopause, as well as associated hormonal and cardiometabolic changes, in ways that are highly dependent on healthcare access and sociocultural context. Variations in the availability and continuity of reproductive and preventive services across healthcare systems may, therefore, have lasting implications for healthy longevity. More broadly, sex and gender may modify how individuals experience mobility-related exposures and respond to preventive interventions across the life course. Future studies should incorporate sex- and gender-sensitive perspectives to better understand these interactions.

Beyond biological differences, mobility-related experiences are embedded within gendered social and occupational contexts. Expatriate relocation may differentially affect social networks, employment trajectories, caregiving responsibilities, and autonomy in healthcare decision-making, potentially amplifying existing health inequalities. These influences may alter access to preventive services, social support, and health-related behaviours across the life course. Consequently, a sex- and gender-sensitive perspective is essential for understanding how mobility-related exposures shape healthy longevity in increasingly diverse and mobile populations.

Within this framework, awareness, knowledge, and motivation mediate how contextual exposures translate into engagement with longevity-oriented interventions. Consequently, mobility-related influences are unlikely to be linear or uniform. Rather, repeated transitions may generate path-dependent trajectories in which the effects of earlier experiences shape subsequent exposures and opportunities. Incorporating mobility into life-course frameworks may therefore provide a more comprehensive understanding of healthy longevity in an increasingly interconnected world.

Several propositions may be derived from the framework. First, repeated geographic transitions may reduce continuity of preventive care and engagement with longevity-oriented interventions. Second, the effects of mobility on healthy longevity may vary according to life stage and the timing of migration. Third, socioeconomic resources, insurance coverage, and legal residency status may moderate the relationship between mobility and health trajectories. Finally, digital health infrastructure may mitigate some of the discontinuities associated with relocation.

## IMPLICATIONS FOR RESEARCH

Viewing expatriates as quasi-experimental populations reframes healthy longevity as a process shaped not only by biological factors, but also by repeated transitions across healthcare systems, sociocultural environments, and physical contexts. From a research perspective, expatriates provide opportunities to examine how healthcare access, social relationships, cultural norms, and environmental exposures interact across the life course to influence health behaviours and engagement with preventive interventions. Such a perspective may facilitate more longitudinal, comparative, and context-sensitive approaches to healthy longevity research.

Future studies may employ longitudinal cohort designs, comparative studies across expatriate subgroups and other mobile populations, as well as mixed methods approaches that capture both quantitative and experiential dimensions of mobility. Potential populations of interest include corporate expatriates, retirees living abroad, international students, return migrants, and other groups experiencing repeated cross-context transitions. The framework may be particularly relevant in health contexts requiring sustained preventive engagement and continuity of care, such as cardiometabolic disease prevention, healthy ageing, and longevity-oriented interventions. Conversely, its applicability may be more limited in geographically stable populations or in contexts where mobility plays a less prominent role in shaping health trajectories.

## IMPLICATIONS FOR HEALTH SYSTEMS

For health systems, increasing global mobility underscores the need for adaptable and interoperable models of care. Contemporary migration is increasingly characterised by repeated movements across origin, destination, and return settings rather than by one-way relocation [7]. Expatriates frequently navigate multiple healthcare ecosystems with differing levels of accessibility, affordability, familiarity, and perceived quality [10]. These differences may contribute to fragmented care, inconsistent insurance coverage, and reduced continuity of preventive services. Such challenges are particularly relevant for longevity-oriented interventions that require sustained engagement across healthcare systems and jurisdictions. Addressing these barriers may require improved data portability, cross-border continuity of care, and culturally responsive service delivery models.

Digital health infrastructure may partially mitigate some of the discontinuities associated with repeated relocation. Telemedicine, wearable health monitoring devices, mobile applications, and online health communities increasingly enable continuity of care and facilitate access to health information across jurisdictions. However, these technologies may also introduce new challenges, including misinformation, health anxiety, and excessive health optimisation behaviours. As digital tools become increasingly integrated into preventive and longevity-oriented care, understanding their moderating effects on mobility-related health trajectories represents an important area for future research.

From a precision public health perspective, incorporating mobility and life-course transitions into risk stratification may enhance the design and implementation of healthy longevity interventions. Standardised approaches based primarily on geographically stable populations may inadequately capture the cumulative and context-dependent exposures experienced by increasingly mobile individuals. Recognising mobility as a life-course determinant may, therefore, support more personalised and context-sensitive preventive strategies.

## ETHICAL AND GOVERNANCE CONSIDERATIONS

Future research involving internationally mobile populations should also explicitly consider ethical and governance dimensions. As mobility increasingly transcends national boundaries, issues of equitable participation, shared responsibility, and appropriate governance of cross-border health data become increasingly important. Research should be conducted using culturally sensitive approaches and ensure that the benefits and burdens of participation are distributed fairly across populations. More broadly, healthy longevity among mobile populations may require collaborative approaches involving individuals, families, healthcare systems, employers, and countries of origin and destination to promote equitable and sustainable access to preventive and longevity-oriented care.

## LIMITATIONS OF CONCEPTUAL FRAMEWORK

This conceptual framework has several limitations. First, the literature cited was selected purposively rather than through systematic or scoping review methods and therefore may be subject to selection bias. Second, the framework may be particularly applicable to expatriate populations with relatively greater socioeconomic resources, such as corporate employees and retirees, and may not fully reflect the experiences of the broader spectrum of internationally mobile individuals. Expatriates themselves are heterogeneous with respect to socioeconomic position, legal status, insurance coverage, and healthcare access, and mobility-related influences on healthy longevity are unlikely to be uniform. Finally, the proposed framework is conceptual, rather than causal, and is intended to generate hypotheses for future longitudinal, comparative, and mixed-method studies across diverse mobile populations and settings.

## CONCLUSIONS

Expatriates represent a growing population whose transnational life courses provide a distinctive lens through which to examine how healthcare systems, cultural environments, and behavioural adaptations interact across time to shape healthy longevity. In an increasingly interconnected world, mobility should be viewed not as an exception to otherwise stable ageing trajectories, but as an important determinant operating across the life course.

Conceptualising expatriates as quasi-experimental populations provides a useful framework for advancing healthy longevity research and implementation in a mobile world. Integrating mobility and life-course transitions into conceptual and empirical models may improve the scientific rigor, contextual relevance, and practical applicability of interventions aimed at extending healthspan. This perspective may also inform future comparative and longitudinal studies examining how mobility modifies ageing trajectories across diverse populations.

Ultimately, incorporating mobility into healthy longevity frameworks may contribute to a more globally relevant understanding of ageing, recognising that health trajectories are shaped not only by biology, but also by dynamic interactions among individuals, environments, and healthcare systems across time.

## Data Availability

**Data availability:** No data sets were generated or analysed during the current study because it presents a conceptual framework developed based on published literature.
